# Cutaneous and pulmonary sarcoidosis following treatment of multiple sclerosis with interferon-β-1b: a case report

**DOI:** 10.1186/1752-1947-7-270

**Published:** 2013-12-13

**Authors:** Mohammad Ali Sahraian, Abdorreza Naser Moghadasi, Mahsa Owji, Mehrdad Maboudi, Farid Kosari, Jeanie C McGee, Alireza Minagar

**Affiliations:** 1 MS Research Center, Neuroscience Institute, Tehran University of Medical Science, Hassan Abad Sq, Tehran, Iran; 2 Department of Pathology, Sina Hospital, Tehran University of Medical Science, Hassan Abad Sq, Tehran, Iran; 3 Department of Neurology, LSU Health Sciences Center, Shreveport, LA 71103, USA

**Keywords:** Interferon-β, Multiple sclerosis, Sarcoidosis

## Abstract

**Introduction:**

Several cases of sarcoidosis following treatment with interferon-α have been reported in the literature, but those following interferon-β are very rare. We report the case of a patient with multiple sclerosis who developed pulmonary and cutaneous sarcoidosis following treatment with Betaseron® (interferon-β-1b).

**Case presentation:**

A 33-year-old Caucasian woman with a history of multiple sclerosis, treated with interferon-β-1b for 2.5 years developed erythema nodosum in her lower limbs, a breast abscess, and unilateral adenopathy of her left lung. A skin biopsy confirmed sarcoidosis. After the discontinuation of interferon-β-1b and treatment with indomethacin and prednisolone, she recovered.

**Conclusions:**

Sarcoidosis is considered one of the most common multiple sclerosis imitators with involvement of the central nervous system. However, although rare, sarcoidosis can develop following treatment with interferon-β-1b and should be considered in patients with multiple sclerosis treated with beta-interferons who develop pulmonary or extra-pulmonary manifestations of sarcoidosis. Interferon-β-1b discontinuation is the first and most important step in the treatment of such cases followed by treatment with corticosteroids.

## Introduction

Sarcoidosis is a chronic inflammatory disease commonly involving granulomatous involvement of different organs [1]. The prevalence of the disease is three to five per 100,000, and the most common age of occurrence is between 25 and 40 years [2]. From an immunopathologic point of view, interferon-γ (IFN-γ) plays an important role in granuloma formation of this disease [3]. Evidence for the role of other IFNs, such as IFN-α and β in the pathogenesis is rarely reported. However, there are increasing reports of sarcoidosis following treatment with IFN-α and IFN-β in different diseases, including hepatitis C, renal cell carcinoma, multiple myeloma, and multiple sclerosis (MS) [4]. We report the case of a patient with MS who developed pulmonary and cutaneous sarcoidosis following Betaseron® (IFN-β-1b) treatment.

## Case presentation

**Figure 1:**
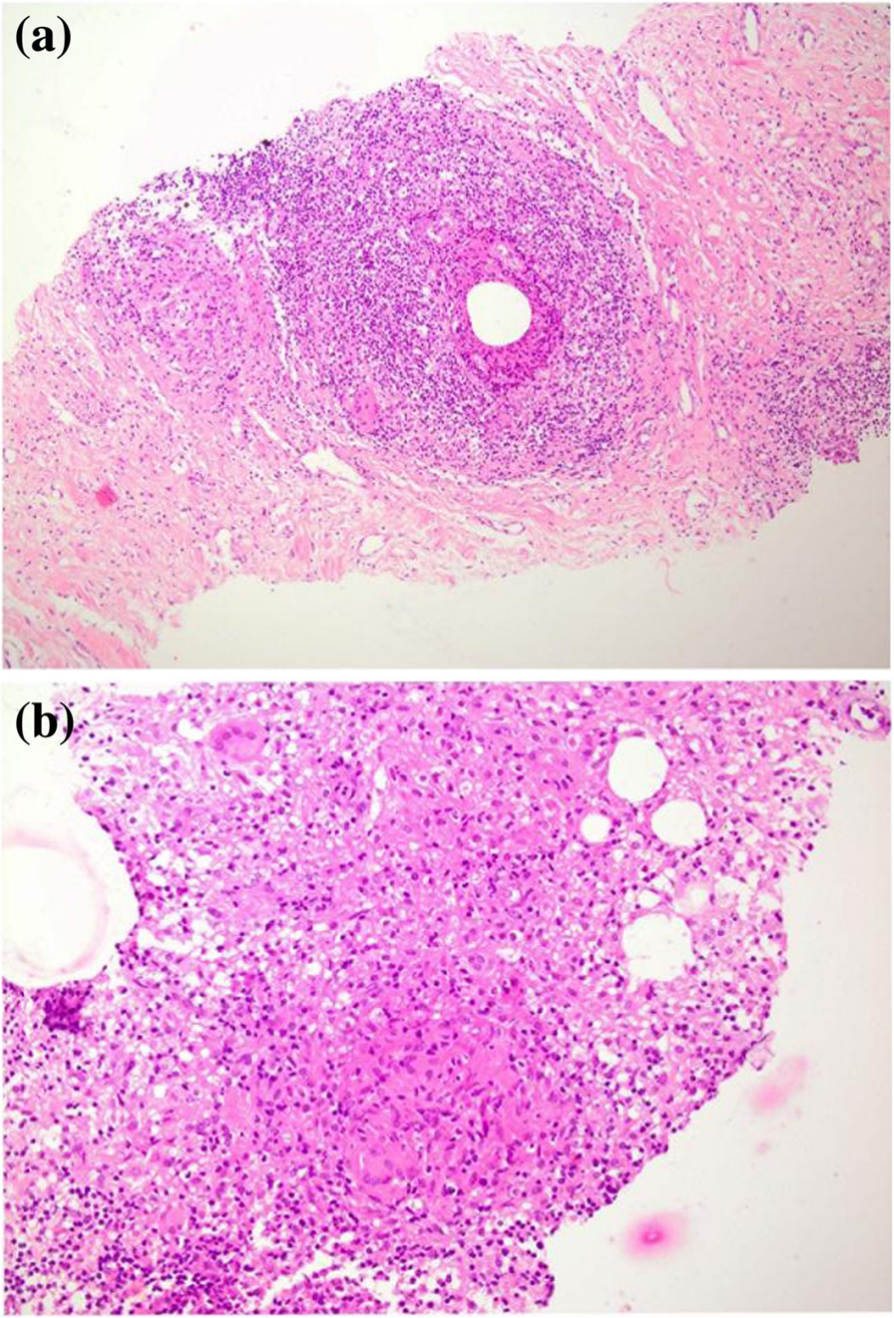
**A section of breast biopsy. (a)** Low power view of the breast lesion showing excess mononuclear infiltration around fat space with a multinucleated giant cell in periphery. Hematoxylin and eosin (×100). **(b)** High power view of a well-formed granuloma composed of aggregation of epithelioid histiocytes with multinucleated giant cells. Hematoxylin and eosin (×200).

A 33-year-old Caucasian woman developed paresthesia in her lower limbs followed by ataxia in 2009. Magnetic resonance imaging of her central nervous system revealed the presence of multiple hyperintense lesions in T2-weighted images of her brain and spinal cord consistent with a diagnosis of MS. Some of the lesions on T1-weighted views showed enhancement following intravenous infusion of gadolinium. A survey for vasculitis, including anti-nuclear antibody (ANA) and anti-phospholipid antibody, was negative and treatment with IFN-β-1b was initiated in the patient. At 2.5 years later and while on IFN-β-1b she developed painful erythematous lesions on the anterior sides of both her legs consistent with erythema nodosum. A week later she developed pain, swelling, and erythema of her left breast, dry cough, chills, and fever. Upon breast examination an erythematous and tender mass with dimensions of 7×7cm was discovered. Her pulmonary examination was normal, and her abdomen did not have organomegaly. Multiple erythema nodosum lesions with dimensions of 4×5cm were observed in both her legs. She had no previous history of trauma to her breast tissue. Laboratory findings revealed hemoglobin of 9.5 and an erythrocyte sedimentation rate of 123. The results of vasculitis tests, including ANA, anti-double-stranded deoxyribonucleic acid (DNA), anti-neutrophilic cytoplasmic antibodies (ANCAs), C3, and C4 were normal. The results of angiotensin converting enzyme, thyroid function tests, Mantoux test, pulmonary function test, and abdominopelvic sonography were also normal. Serum and urine calcium levels as well as X-rays of the bones of both her hands were normal too. A computed tomography scan of her chest revealed unilateral hilar adenopathy of her left lung. A bronchoscopy was performed but was normal. Breast sonography showed a 5×2cm abscess with inflammation in its peripheral fat. Following the drainage of her breast abscess, the specimen was sent for histopathologic examination. In sections taken from the specimen, multiple granulomas composed of epithelioid histiocytes and mononuclear infiltration with multinucleated giant cells were observed, which generally suggested granulomatous mastitis (Figures 1a and 1b). The results of bacterial and fungal and mycobacterial cultures from the abscess were negative. An acid-fast bacillus (AFB) stain was negative as well. Based on the findings of unilateral hilar adenopathy of her left lung and the presence of erythema nodosum and granuloma in the biopsy, she was diagnosed with sarcoidosis. A comprehensive review of the literature revealed that both IFN-β and IFN-α may induce sarcoidosis [4]. Thus, IFN-β-1b was discontinued in the patient and glatiramer acetate was begun. She was also treated with indomethacin and prednisolone, and she improved in 4 weeks.

## Discussion

Granulomatous mastitis has several etiologies. In general, the etiology of this disorder can be divided into two major groups including infectious and non-infectious causes. Infectious causes are the main cause of granuloma formation in the human body [5] and granulomatosis can be caused by mycobacterial, fungal and bacterial infections. Non-infectious causes of granulomatosis mastitis are sarcoidosis, Wegener granulomatosis, and the reaction of a breast to a foreign body [6]. Erythema nodosum is usually idiopathic. However, there are other possibilities which may result in this non-specific cutaneous manifestation including beta-hemolytic streptococcal infections, mycobacterial infections, sarcoidosis, inflammatory bowel disease (Crohn's disease and ulcerative colitis) and carcinoma (mainly lymphoma or leukemia) [7].

As mentioned above, the specimen cultures of bacterial, fungal and mycobacterial infections were negative; the results of the AFB stain and Mantoux test were negative as well. In addition, the solid response to treatment with prednisolone points towards a non-infectious etiology. There was no evidence of any form of malignancy in the pathological examination. Since ANCA was normal, Wegener granulomatosis disease was excluded. The patient did not have any gastrointestinal complaints and inflammatory bowel disease is not a usual cause of granulomatous mastitis.

The cutaneous manifestation of sarcoidosis is divided into two different types: specific and non-specific [8]. As granulomas are seen on biopsy, the sarcoid skin manifestation will be entitled specific cutaneous sarcoid lesions [8]. In our case, granulomas were detected on breast biopsy. So, the breast abscess of our patient is a specific kind of sarcoid lesion. However, erythema nodosum is recognized as the most common non-specific skin manifestation of sarcoidosis [8]. Hence, it can be said that our case had both specific and non-specific cutaneous sarcoid lesions. To the best of our knowledge, only seven patients with sarcoidosis following IFN-β therapy have been reported in the literature. The initial report by Abdi *et al*. in 1987 reported the case of a 57-year-old woman with renal cell carcinoma who developed pulmonary sarcoidosis following treatment with IFN-β and vinblastine [9]. Subsequent reports include a case report by Bobbio-Pallavicini *et al*. in 1995 which discusses a woman diagnosed with multiple myeloma who developed sarcoidosis involving different organs, including liver, bone, and lungs following treatment with IFN-β [10]. Mehta *et al*., in 1998, reported the case of a 57-year-old man with MS who developed cutaneous sarcoidosis after being treated with IFN-β for 2 years and 2 months [11]. More recently, in 2005, O'Reilly *et al*. reported the case of another patient with pulmonary sarcoidosis following weekly treatment with IFN-β [12]. Since that time, three other patients have been reported, all published in 2012. Of these three patients, one was a 39-year-old woman with skin and pulmonary involvement after 3 years of IFN-β treatment, the second one was a 35-year-old man who developed pulmonary sarcoidosis after 6 years of IFN-β treatment, and the third one was a 30-year-old woman with a 5-month history of IFN-β treatment who also had only pulmonary sarcoidosis [13-15]. The present patient was a 33-year-old Caucasian woman with MS who was treated with IFN-β for 2.5 years and developed both pulmonary and cutaneous sarcoidosis. From a clinical standpoint, in all seven reported patients, IFN-β was discontinued after the discovery of the adverse developments. The first patient was not treated [9]. However, the second patient was treated with corticosteroids, and the third patient recovered following treatment with a combination of hydroxychloroquine, psoralen, and ultraviolet A [10,11]. One patient improved without any treatment and one was treated with prednisone, hydroxychloroquine, and methotrexate [8,9]. The final two reported patients were treated with prednisolone and hydroxychloroquine due to the continuity of the signs and complaints, and prednisolone, respectively. In the last patient, prednisolone resulted in complete resolution [12,13]. Treatment of our patient with a combination of indomethacin and prednisolone caused a significant decrease in the signs and symptoms. A total of eight patients, including the current presented patient, have been reported to have sarcoidosis following treatment with IFN-β, six of which were patients with MS. The average duration of treatment with IFN-β was 28.1 months. In a considerable presentation, the rate of pulmonary and skin involvement in these eight patients was 87.5% and 37.5%, respectively. While IFN-β-induced sarcoidosis may appear to be a separate entity, further research should be conducted to determine factors involved in the occurrence of sarcoidosis following IFN-β treatment. The development of sarcoidosis following treatment with IFN-β seems evident; however, the event may be attributed to its immunomodulatory properties or its effect on the activation of proinflammatory mediators, which can result in the formation of granuloma [13]. The diagnosis of IFN-induced sarcoidosis (IIS) in a patient with MS may be questionable since it is difficult to establish a clear-cut causal relationship. It is well known that sarcoidosis is a great imitator of MS [16]. Therefore, views that the patients presented in this report had from the beginning only sarcoidosis and not MS should be taken into consideration. However, it should be stressed that the available data on the ratio of involved organs in the patients with IIS are different from natural sarcoidosis [4], so it is most probable that IFN-β, similar to IFN-α, can cause sarcoidosis in patients with MS due to its immunomodulatory properties. Further, it should be noted that the immunopathology of MS may actually contribute to the induction of sarcoidosis in patients undergoing IFN-β treatment. In general, this phenomenon is a form “biologics-induced autoimmune disease”, which has been recently described by Perez-Alvarez *et al*. and includes diverse autoimmune diseases caused by different biological drugs [17].

## Conclusions

Sarcoidosis is one of the differential diagnoses of MS. The present case demonstrates that treatment with IFN-β-1b can result in the development of sarcoidosis in patients with MS. Therefore, in patients with respiratory or cutaneous symptoms, IFN-β-induced sarcoidosis should be considered among differential diagnoses. Cases of IFN-β-induced sarcoidosis are therapeutically responsive to the discontinuation of the IFN-β and treatment with corticosteroids.

## Consent

Written informed consent was obtained from the patient for publication of this case report and accompanying images. A copy of the written consent is available for review by the Editor-in-Chief of this journal.

## Abbreviations

AFB: Acid-fast bacillus; ANA: Anti-nuclear antibody; ANCA: Anti-neutrophilic cytoplasmic antibodies; IFN: Interferon; IIS: IFN-induced sarcoidosis; MS: Multiple sclerosis.

## Competing interests

The authors of this case report declare that they have no competing interests.

## Authors’ contributions

MAS, ANM, MO, MM, and FK prepared the text and collected all the medical data, reviewed the literature, provided suitable references, and assisted in the draft version of the paper. MAS and FK reviewed and interpreted magnetic resonance imaging and histopathology reports, collected clinical data, and assisted with preparation of the manuscript. JM and AM reviewed the paper and revised it to the final format and re-wrote parts of the paper. All authors read and approved the final manuscript.
